# Role of amyloid precursor protein (APP) in regulation of neuronal genes

**DOI:** 10.1186/2193-1801-4-S1-L40

**Published:** 2015-06-12

**Authors:** Natalia N. Nalivaeva

**Affiliations:** I.M. Sechenov Institute of Evolutionary Physiology and Biochemistry RAS, Russian Federation, St. Petersburg, 19423 Russia; School of Molecular and Cellular Biology, Faculty of Biological Sciences, University of Leeds, UK

**Keywords:** Amyloid precursor protein, neprilysin, histone decetylases

Understanding the mechanisms regulating gene expression in the course of development and adaptation of the organism to the permanently changing environment and in the case of pathology is fundamentally important. Special attention in recent years has been paid to the processes of epigenetic regulation of neuronal genes involving chromatin modifications at the level of DNA methylation and histone acetylation. In these processes an important role belongs to the histone deacetylases (HDAC) which control gene silencing. Recently it was shown that the amyloid precursor protein (APP) and its intracellular domain (AICD) participate in regulation of expression of a number of neuronal genes, including those involved in amyloid metabolism and clearance. The list of APP-regulated genes includes the major amyloid-degrading enzyme neprilysin (NEP), a transport protein transthyretin (TTR), aquaporin and others. Our studies strongly indicate that AICD regulation of NEP and TTR is APP isoform-dependent and cell-type specific. In this process AICD competes for gene regulation with HDACs and treatment of cells or animals with HDAC inhibitors such as valproic acid results in up-regulation of NEP and TTR mRNA and protein levels and increased NEP activity leading to a reduction in total cellular amyloid (Aβ) peptide levels. Regulation of other proteins, e.g. acetylcholinesterase, does not involve AICD but requires full length APP molecules. APP overexpression in neuronal cells was also shown to affect the levels of HDAC gene products which might explain its role in gene regulation. Further studies of the APP interactome are important for better understanding of its role in brain development and functioning and for designing the drugs protecting the brain against neurodegeneration, in particular Alzheimer’s disease.

